# The Microbiological Quality of Concentrates for Horses—A Retrospective Study on Influencing Factors and Associations with Clinical Symptoms Reported by Owners or Referring Vets

**DOI:** 10.3390/vetsci9080413

**Published:** 2022-08-05

**Authors:** Sandra Intemann, Bernd Reckels, Dana Carina Schubert, Petra Wolf, Josef Kamphues, Christian Visscher

**Affiliations:** 1 Institute for Animal Nutrition, University of Veterinary Medicine Hannover, Foundation, 30173 Hannover, Germany; 2 Institute for Nutrition Physiology and Animal Nutrition, Faculty of Agricultural and Environmental Sciences, University of Rostock, Justus-von-Liebig-Weg-6b, D-18059 Rostock, Germany

**Keywords:** feed hygiene, horse feeding, microbiology, horse health

## Abstract

**Simple Summary:**

Poor feed hygiene is also referred to as spoilage and can be measured by means of determining growth of microorganisms (bacteria, mold and yeast). Evidence has been provided of mold spores triggering equine asthma, a chronic, non-infectious respiratory disease. Furthermore, a high yeast load is suspected to trigger gastrointestinal disorders (colic) in horses. One aim of the present study was to clarify the possible connection between certain equine diseases and poor feed hygiene. For this purpose, archived reports of hygiene examinations of concentrates for horses were processed statistically. Reports contained information on disease symptoms as reported by horse owners or equine practitioners, as well as results of sensory and microbiological analyses. Further assistance to horse owners and equine practitioners should be provided in evaluating health hazards emanating from poor feed hygiene. Another objective was to assess the possibility of detecting hygiene deficiencies by means of simple examination methods such as determination of dry matter content and sensory analysis in order to estimate the validity of these field methods. It was shown that a connection can be made between mold content of oats and coughing in horses, whereas no connection could be found between poor feed hygiene and equine colic or elevated liver enzyme activities. No significant predictability of poor feed hygiene by means of sensory analysis could be established, whereas a significant association between low dry matter content and mold contamination in grains was shown.

**Abstract:**

Evidence has already been provided that feed-borne mold spores and endotoxins can trigger chronic, non-infectious respiratory disease if inhaled. Furthermore, deficiencies in feed microbiology are suspected to trigger gastrointestinal and liver disorders in horses, but the connection needs further clarification. Most of the previous studies regarding horse feed hygiene focused on forage, whereas research regarding hygienic quality of concentrates is scarce. In the present study, results of reports on hygienic quality of compound feed and cereals for horses were evaluated secondarily. Results included sensory findings, and counts of aerobic bacteria, molds and yeasts determined by cultivation and lipopolysaccharide (LPS) contents. It was found that microbial counts of compound feed exceeded VDLUFA orientation values significantly more frequently than cereals (38.4 vs. 22.6%). However, average counts of bacteria, molds and yeasts were higher in cereals than in compound feeds (*p* < 0.0001, respectively). Mold counts in grains were significantly higher if dry matter contents were below 86% (*p* = 0.0201). No relation could be established between the anamnestically reported gastrointestinal disorders or elevated liver enzyme activities and microbiological deviations. Mold counts of concentrates which were suspected to cause coughing in horses were significantly higher than mold counts of control samples (3.29 vs. 2.40 log^10^ cfu g^−1^, *p* = 0.0313). These results indicate that hygienic status of concentrates is relevant for horse health in the respiratory tract.

## 1. Introduction

In horse feeding practice, forage-based rations are complemented by concentrates to supplement performance requirements [1]. Individual feedstuffs such as cereals or compound feed in pelleted or non-pelleted (e.g., “muesli”) forms can be used for this purpose [2]. Cereals are used to meet the energy requirements of horses, whereas compound concentrates may also contain protein-rich ingredients (e.g., soya, peas, beans) and mineral supplements [3]. Compound feedstuffs are mostly produced and marketed industrially [4], whereas cereals such as oats are mostly obtained directly from agricultural production [5].

In the literature, it was already reported that inadequacies in the hygienic quality of basic and concentrated feeds for horses are frequent [6,7,8]. To assess the hygienic quality of feedstuffs, sensory and microbiological methods are applied [9]. Feedstuffs are checked for abnormal odor, color and texture and whether storage pests have infested the feed. This procedure can give indications of inadequate microbiological quality, especially of molds and yeasts [7,10]. In terms of quantifying the extent of microbial growth, microbiological methods such as cultivation are used. Plate counts are then expressed in colony forming units (cfu) per gram sample. For evaluating the microbiological quality of horse feed, different reference values for plate counts of bacteria, molds and yeasts are used in the literature. Sliwinsky et al. [8] used reference values from a study conducted in 1991 by Schmidt [11], who considered germ counts above 2 × 10^6^ cfu and mold counts above 1 × 10^4^ cfu inadequate. Kaya et al. [12] used a benchmark of 10^4^ cfu per gram sample for yeasts, 70–10^3^ cfu per gram for mold and 10^6^ cfu per gram sample for bacteria, as proposed by Kamphues in 1999 [13]. The Association of German Agricultural Investigation and Research Institutions (VDLUFA) [14] established orientation values in 2017 to indicate to which extent the microbial load can be considered as normal. For this purpose, molds are classified into “product-typical” and “spoilage” or “storage” molds and distinct orientation values were established for both groups [15]. The same was applied to bacteria, whereas yeasts have not been further classified [14]. In the present study, VDLUFA orientation values were used to assess microbial counts as inadequate or excessive.

Measures preventing the spoilage of feedstuffs are referred to as feed hygiene according to EU legislation [16]. For example, reducing the initial content of microorganisms in feedstuffs can improve storage stability [17]. Therefore, hydrothermal processes such as pelleting can be used [18]. As another measure of feed hygiene, the moisture content of concentrates has to be kept at a maximum of 14% due to dry conditions at harvest or the subsequent drying of grains in order to impair growth conditions of microorganisms [17]. If grain is stored at moisture contents above 14.5%, spoilage molds will proliferate in the material, resulting in heating and dry matter losses [19]. As for the growth of product-typical molds such as *Alternaria, Cladosporium* or *Fusarium*, moisture contents of 20–25% are crucial, whereas storage molds can proliferate at markedly lower moisture contents of 14–18%. The main factors that favor mycotoxin production in stored grain have shown to be high grain moisture content (16–30%), high grain temperature (25–32 °C) and high relative humidity of air in storage facilities (80–100%) [20]. Hence, as well as ensuring cleanliness of facilities and pest control [17], feed hygiene includes measures to keep air humidity levels below 70% [21] during storage.

If excessive microbial growth occurs in feedstuffs, this spoilage process yields the decomposition of the feed, and thus, nutrient losses [22]. In addition, the sensory properties of the feed, such as odor and palatability, are negatively affected, which can lead to reduced feed intake and feed refusal [23]. As for excessive mold growth, the production of mycotoxins in stored grains is problematic, with potential health hazards posed to animals and humans [24]. Field fungi of the genus *Fusarium* colonize feedstuffs mainly pre-harvest, whereas spoilage molds such as *Penicillium* and *Aspergillus* proliferate during storage [25]. Production of mycotoxins can occur in feedstuffs colonized by molds under certain conditions of stress impacting the fungi, such as changing environmental conditions, shortage of substrates and the competing growth of other microorganisms [26]. In temperate climates, mycotoxins produced mainly pre-harvest such as deoxynivalenol (DON, produced by *Fusarium graminearum* and *Fusarium culmorum*), as well as T-2 and HT-2 toxins (*Fusarium langsethiae*), and mycotoxins produced pre-harvest such as zearalenone (*Fusarium graminearum* and *Fusarium poae*) [27] are of major interest when it comes to minimizing natural contaminants in the food chain [19].

Regarding the effects of mycotoxins on horse health, T-2 and HT-2 have been shown to induce central nervous disorders [28] and zearalenone may alter the reproductive function in mares [29]. Regarding the reduction in feed intake due to ingestion of DON, horses are considered to have a higher tolerance for this toxin than other species [30].

Previous studies investigated the hygienic quality of concentrates and found that frequencies of inadequacies were different between concentrate types. In the study by Sliwinsky et al. [8], 64% of the oat samples were found to have greatly increased counts of bacteria of above 1.2 × 10^7^ cfu per gram as fed, whereas this applied to 28% of “bought-in” compound feed. As for molds, counts were determined above 2 × 10^5^ cfu in 20% of oat samples, but none were above for the compound feed samples. No distinction was made between product-typical and spoilage molds or bacteria. Wolf et al. [7] used orientation values of the VDLUFA from 2002 [31] to assess microbiological quality of feeds for horses. They found deficiencies in 34% of the grain and 9% of the industrially produced feed samples.

Regarding effects of inadequate feed hygiene on horse health, different health disorders are discussed, for example, regarding the gastrointestinal and respiratory tract [32,33,34].

In equine medicine, the term colic stands for a generic form of abdominal pain that usually originates in the gastrointestinal tract and is one of the most frequent causes of emergency treatment by equine practitioners and death [35,36]. This painful state can be induced by different processes; for example, by distension of the intestinal tube due to congestion of ingesta or excessive gas formation [37]. Alimentary causes of colic have already been verified, such as excessive intake of concentrates [36,38] regardless of the hygienic quality of the ingested material. The intake of feed of inferior hygienic quality is also suspected of causing gastrointestinal disorders, since the intake of material rich in carbohydrates may favor conditions for microbial growth in the gastrointestinal tract. In the equine stomach, pH values can stay above 5.5 for 2–3 h after feed intake [39]. Thus, feed borne microorganisms may subsequently reach the small intestine [39] and lead to gas formation [40]. This process was particularly described for feed-borne yeasts [41]. Furthermore, the function of the enteric nervous system, and thus, intestinal peristalsis, can be disturbed by an excessive microbial content in the feed [42]. In a case study, five of six horses showed colic symptoms after ingesting oats highly contaminated with molds [43].

In terms of investigating the effect of feed hygiene on respiratory equine diseases, aeroallergens such as lipopolysaccharides (LPSs) and mold spores have been a focus of several studies. Due to excessive growth of Gram-negative bacteria, the endotoxin LPSs as a cell-wall component of these microbes can accumulate in the feed [44]. LPSs play a role as aeroallergens in triggering chronic, non-inflammatory lung diseases in horses [45]. This is also referred to as equine asthma (EA) [46], which is a reversible bronchial hyperreactivity triggered by environmental allergens characterized by symptoms such as dyspnea and chronic coughing, and can be compared to asthma in humans [47]. The prevalence is estimated at 10–20% in the Northern Hemisphere [24]. In Switzerland, in a randomized study it was found that as many as 50% of the horse population may be affected [48]. Additionally, relevant for this disease complex is the content of inhalable dust as well as its content of mold spores, especially of the genus *Aspergillus* [49,50,51]. Exposure to endotoxins, forage mites and mold spores from dusts from the provided roughage has been experimentally shown to induce symptoms of EA [45]. Vandenput et al. [50] showed that rolled grains can be a source of mold-contaminated feed dust. Whole grains as well as industrially produced concentrates were of minor importance with regard to the emission of aeroallergens.

Scientific knowledge on the effects of microbial contamination of feeds on liver function in horses is scarce. Hepatic necrosis was observed in horses affected by aflatoxicosis [52]. In cattle, symptoms of hepatogenic photosensitization were observed after the ingestion of moldy hay [53]. Moreover, elevated activity of liver enzymes was observed in a dairy herd after feeding on silage contaminated with molds [54]. More evidence of a possible association between the mold contamination of feedstuffs and liver disease in horses would be desirable.

Considering the potential health hazards of the hygienically deficient feeds listed above, it is recommended to critically examine the hygienic status of the feeds used [7,17,55].

The aim of the present study was to obtain further information on the potential effects of feedstuffs with microbial contents exceeding orientation values for usual quality as set by the VDLUFA [14] on horse health. For this purpose, differences in microbial counts of different concentrate types were investigated in order to gain further insights into the hygienic quality of different concentrate types. Another focal point of the research study was to clarify whether microbiological deviations can be predicted by means of sensory analysis. Furthermore, the objective was to clarify whether the occurrence of microbiological deviations in feedstuffs is to be expected if clinical symptoms such as gastrointestinal disorders occurred in horses after feeding of the material.

## 2. Materials and Methods

For the purpose of this study, data of examination reports on hygienic quality of concentrates that were submitted to the Institute for Animal Nutrition at the University of Veterinary Medicine Hannover, Foundation, Hannover, Germany between 1993–1999 (period 1) and 2010–2016 (period 2) were analyzed retrospectively. Reasons for the submission of feed samples for microbiological examination included the routine checking of feed hygiene or feedstuffs suspected of inducing disturbances or disease symptoms due to insufficient hygienic quality.

In order to investigate the hygienic quality, different methods were applied at the Institute for Animal Nutrition. The dry matter (DM) content was determined and feedstuffs were examined sensorily in a standardized manner. Microbiological examination was then performed at the Institute for Microbiology of the University of Veterinary Medicine Hannover, Foundation to determine counts of aerobic bacteria, molds and yeasts, and thus, quantify the microbial load. In the first examination period, determination of LPS content was performed to investigate endotoxin levels in concentrates. The methods of the procedure mentioned above are described in the following subsections.

### 2.1. Examination Methods

Information derived from the report included the type of concentrate, the time period of submission, the preliminary report by the sender of the material, the dry matter content and, if analyzed, the LPS content. Feedstuffs investigated in this study were cereals and compound feedstuffs for horses. The preliminary report included the reason for submission and, where indicated, provided information on symptoms that occurred in horses after feeding of the material. Due to the retrospective design of the present study, no standardized clinical examination of horses could be performed. Reported symptoms were observed by senders (veterinary practitioners or horse owners). If feed samples were submitted for routine examination, it was presumed that no clinical symptoms were observed in horses after feeding.

Sensory deviations were acquired in a qualitative manner following the scheme for sensory analysis according to Kamphues [55] (see Table 1).

For determination of dry matter (DM) contents, the feed samples were dried at 103 °C until weight constancy was achieved [56]. The DM content should be at least 86% [17,57], whereas less than 86% was considered inadequate.

Microbiological cultivation was performed to determine counts of aerobic bacteria, molds and yeasts. The sample material was first diluted in peptone water. Until 31 October 2012, the sample mass amounted to 25 g and the solution volume was 225 mL. From November 2012 onwards, 20 g of the sample and 380 mL of nutrient solution were used. Bacteriological cultivation was performed on blood agar and Gassner plates. Cultivation on Gassner plates was only performed if *Enterobacteriaceae* was at issue from November 2012 onwards. For the cultivation of molds and yeasts, a fungal culture medium with antibiotic additive produced at the Institute of Microbiology of the University of Veterinary Medicine Hannover, Foundation (“Hamburger Testagar”) was used [58]. The dilution was spread directly and in a dilution series on the abovementioned plates. Dilution was performed in intervals of 10 with ranges of 10^−4^ to 10^−8^ (aerobic bacteria) and 10^−2^ to 10^−5^ (yeasts and molds), respectively [58].

For bacteriological cultivation, samples were incubated for two days at 37 °C, whereas incubation temperature for yeasts and molds was 30 °C and duration was five to seven days. For the determination of the plate counts, slides with approximately 50 colonies were taken into account. The plate count was designated as below the detection limit, “below 10^5^” for bacteria and “below 10^3^” for yeasts and molds, if the direct spread was the only plate containing colonies. In cases where a germ count was determined, biochemical methods were applied to differentiate the predominant bacteria. The identification of fungal species was carried out microscopically, whereas yeast species were not further differentiated.

### 2.2. Evaluation of Examination Results

For evaluating the resulting plate counts, orientation values in accordance with the Association of German Agricultural Investigation and Research Institutions (VDLUFA) [14] were used (see Table 2). No orientation values were available for non-pelleted compound feedstuffs for horses. [14] Thus, non-pelleted compound feeds were evaluated using the orientation values for pelleted feed, as proposed in the VDLUFA methods book [40].

The orientation values represent the upper limit of a usual microbial content of various feedstuffs. These were developed for different types of feeds. Particular values are stated for counts of spoilage-indicating and product-typical bacteria and molds, whereas for yeasts, a total count is given.

If counts of aerobic bacteria, molds and yeasts were determined below the detection limit (below 10^5^ cfu for bacteria and below 10³ cfu for molds and yeasts), the values were included into the calculations as half of the detection limit.

Concentrates were classified into the categories “compound feed” (pelleted and non-pelleted) and “cereals” (oats and other cereals). As proposed by the VDLUFA [14], non-pelleted compound feedstuffs for horses were assessed using the orientation values for pelleted compound feedstuffs. Concentrates other than these were excluded from statistical analyses due to the lack of orientation values for other feedstuffs.

The data from the results of sensory analyses were collected according to the scheme of Kamphues [55] (see Table 1). Concentrates were scored and allocated to the categories “no deviations”, “deviations in one category”, “deviations in two to three categories” and “deviations in at least four categories”. Furthermore, deviations that were mentioned in at least ten percent of the cases were subjected to calculations regarding association and agreement with microbiological deficiencies.

### 2.3. Statistical Analyses

Descriptive and analytical statistics were performed using SAS^®^ Enterprise Guide^®^ 7.1 for Windows. Chi-square tests and Fischer’s exact tests were performed to test the association of deviating dry matter contents and microbiological deviations. Table analysis was used to check whether the frequency of microbiological deviations was different between concentrate types. Associations between the occurrence of health disorders, such as colic, coughing, elevated liver enzymes and poor performance, and deviating microbial counts were investigated by table analysis as well. For these analyses, samples submitted for routine examination were considered as the control group. The agreement of examination results of sensory and microbiological analyses was investigated by calculating Cohen’s kappa (ĸ). Mean comparisons were performed to investigate differences between feedstuffs, time periods and preliminary reports. For this purpose, the t-test for normally distributed data and the Wilcoxon rank-sum test for non-normally distributed data were used.

## 3. Results

**Figure 1:**
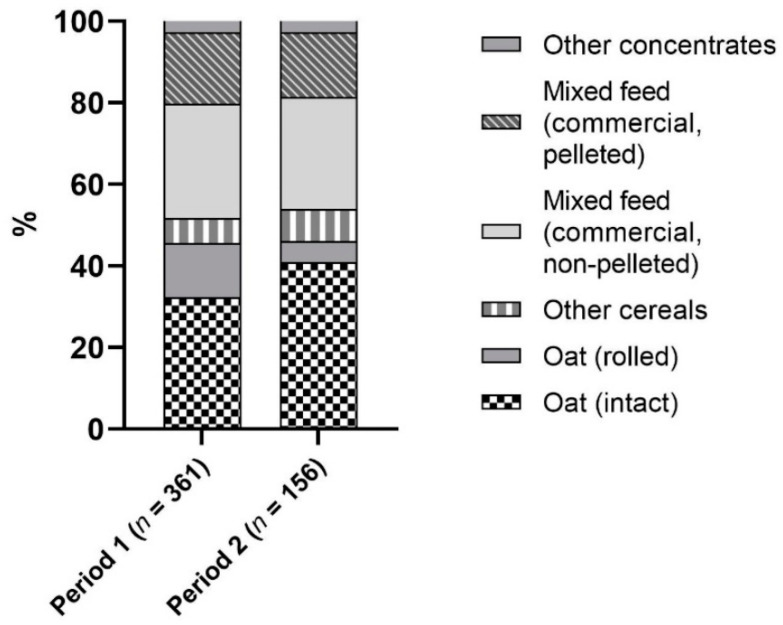
Proportional frequency of feedstuffs examined. Other concentrates (*n* = 27): dried pulp (*n* = 8), carrots (*n* = 5), bran (*n* = 4), milled supplementary feed (*n* = 3), linseed (*n* = 3), maize (*n* = 3), milled linseed (*n* = 1).

The data available consisted of 517 concentrate samples which were analyzed between 1993–1999 and 2010–2016. Regarding the frequency of feed types investigated, oats accounted for the largest share, 45.8% (Figure 1).

The proportion of feed types did not significantly differ between the two investigation periods. Regarding oats, the share of rolled oats decreased significantly (*p* = 0.0027) from 29.1% to 11.1% of oat samples.

**Figure 2:**
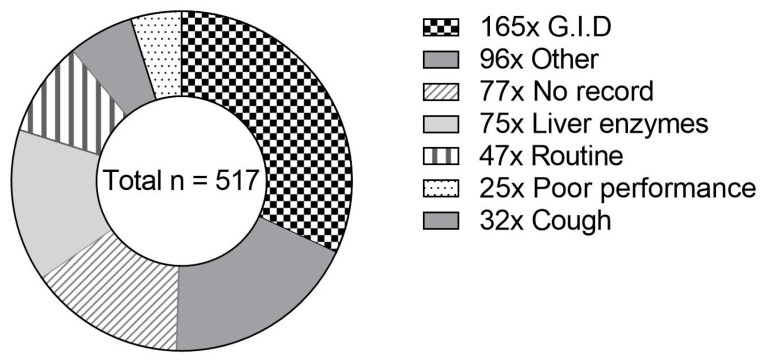
Proportional frequency of preliminary records given by horse owners for submitted concentrate samples (*n* = 517). GIDs—gastrointestinal disorders (*n* = 165): colic (*n* = 150); diarrhea (*n* = 11); watery stools (*n* = 4). Other (*n* = 96): allergy (*n* = 20); suitability (*n* = 15); poisoning symptoms (*n* = 12); abortion (*n* = 9); refusal of feed (*n* = 8); sudden death (*n* = 7); laminitis (*n* = 5); skin problems (*n* = 4); esophageal obstruction (*n* = 4); swollen legs (*n* = 3); alteration of blood counts (*n* = 2); central nervous symptoms (*n* = 2); fungal infection (*n* = 2); permanent heat (*n* = 1); dysfunction of kidney (*n* = 1); muscle stiffness (myogelosis, *n* = 1).

The feedstuffs investigated were submitted to the Institute for Animal Nutrition for various reasons, as displayed in Figure 2.

Gastrointestinal disorders were mentioned most frequently in preliminary records (32%, see Figure 2). In 15% of the cases, no preliminary record was given. A total of 75 samples (14%) were examined because elevated activity of liver enzymes was determined in blood samples after the material was fed. The proportion of samples submitted for routine examination was 9%, whereas this share was 6% for samples that were examined to clarify the cause of coughing. Reasons for submission did not differ significantly between the feed types (*p* = 0.1939).

### 3.1. Proportional Frequency of Deficiencies

**Figure 3:**
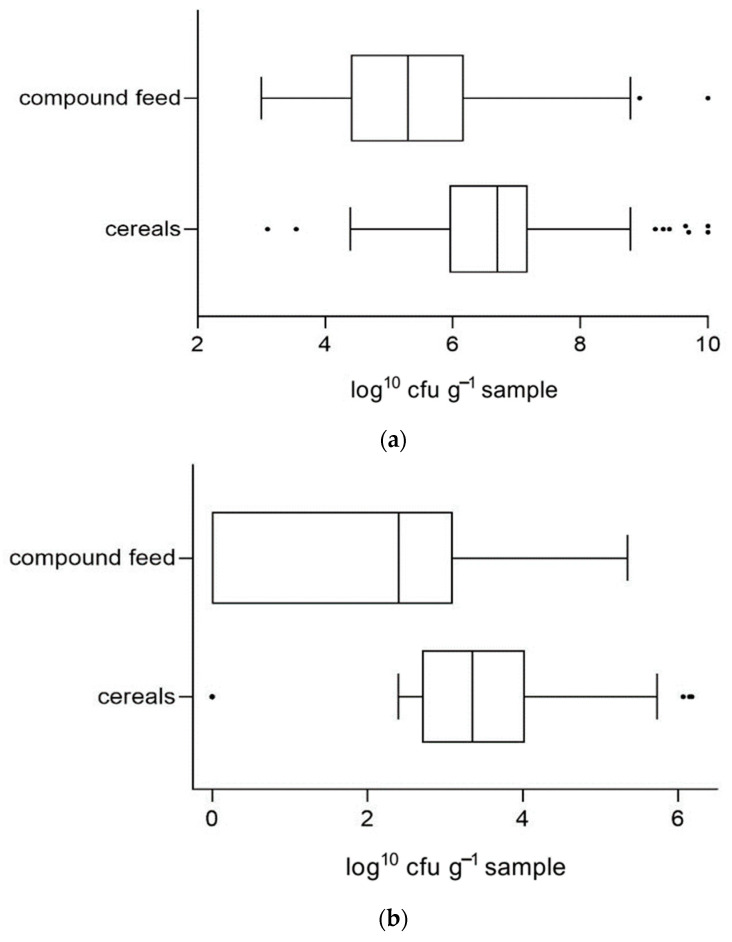

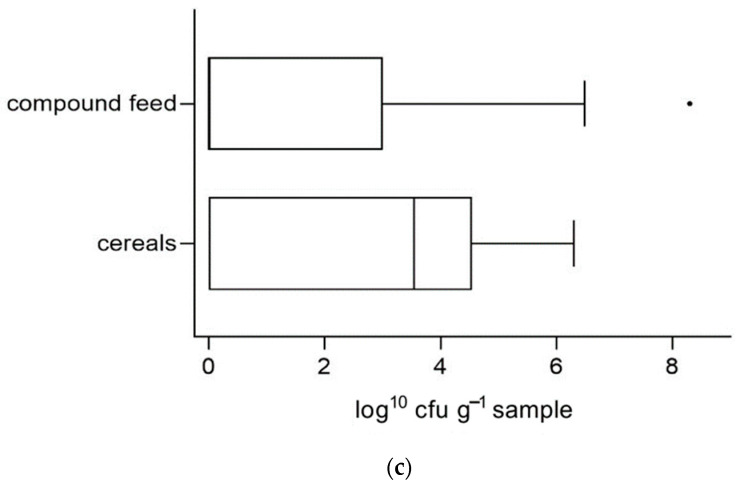
(**a**) Distribution of aerobic germ counts (log^10^ cfu g^−1^ sample as fed) in grains and compound feedstuffs. (**b**) Distribution of mold counts (log^10^ cfu g^−1^ sample as fed) in grains and compound feedstuffs. (**c**) Distribution of yeast counts (log^10^ cfu g^−1^ sample as fed) in grains and compound feedstuffs.

As stated above, feedstuffs were examined by cultural methods whose results concerning aerobic bacteria, molds and yeasts were reproduced in colony forming units per gram sample. Average values of microbial counts (log^10^ cfu g^−1^) in the categories “cereals” and “compound feed” are shown in Figure 3a–c.

Average counts of aerobic bacteria, molds and yeasts were significantly higher in grains than in compound concentrates (*p* < 0.0001 each).

Additionally, the results of microbiological analyses were assessed in accordance with VDLUFA orientation values [14] for the particular feed types (grains or pelleted mixed feeds for horses). The results concerning microbiological deviations plus the proportion of samples with insufficient dry matter content are shown in Table 3.

The microbiological quality of grains was inadequate in 22.6% of the analyzed feedstuff, whereas this applied to 38.4% of compound feeds. Aerobic germ counts of grain samples exceeded VDLUFA values significantly less frequently (*p* < 0.0001) than aerobic germ counts of compound concentrates. The frequency of deviating yeast counts was also higher in compound feeds than in grains (*p* < 0.0001), whereas this did not apply to molds (*p* = 0.0940).

The types of grains (oats, rolled oats, other grains) did not differ significantly regarding the proportion of samples with exceeding microbial counts. Average counts, on the other hand, were significantly higher in rolled oats than in intact oats regarding counts of aerobic bacteria (*p* = 0.0003) and yeasts (*p* = 0.0260).

Microbial counts were assessed as inadequate significantly more frequently in compound feeds that were non-pelleted than in pelleted compound feeds (45.1% vs. 20.5%; *p* = 0.0055). Counts of aerobic bacteria and yeasts exceeded VDLUFA values significantly more frequently in non-pelleted compound feeds than in concentrates that were pelleted (*p* = 0.0174 and *p* = 0.0444), whereas deviation frequency regarding molds was not significantly different. Average counts of aerobic bacteria, molds and yeasts were not significantly different in pelleted and non-pelleted compound feed.

Inadequate mold counts were most frequently caused by exceeding counts of spoilage-indicating molds (93.3% of deviations in grains and 66.6% of deviations in compound feeds). In contrast to this, exceedances of orientation values for aerobic bacteria occurred in the majority of imperfect grain (65.8%) and compound feed samples (68.9%) due to field-typical bacteria.

### 3.2. Comparison of Microbiological Quality in Two Investigation Periods

Examination results of the two investigation periods were compared regarding average counts of aerobic bacteria, molds and yeasts, as well as regarding the proportional frequency of plate counts exceeding the VDLUFA criteria [14] (Table 4).

Regarding molds, average counts in grain samples increased from the first to the second investigation period (*p* = 0.0360), whereas the proportion of samples with counts exceeding VDLUFA values did not change significantly (*p* = 0.5690, Table 4). Neither average yeast counts nor the proportion of samples with inadequate yeast counts was significantly different between both periods. In contrast to this, a significant decrease could be shown in average counts and deviation frequency of aerobic bacteria (*p* = 0.0003 and *p* = 0.0243, respectively).

Although the overall quality of grain samples differed as listed above, no significant changes could be observed at the level of the individual grain types (oats, rolled oats and other grains).

Microbiological quality of compound feed did not differ significantly between both investigation periods regarding average plate counts and proportional frequency of deviations. For this reason, the results are not presented here for compound feeds, as is the case for cereals (Table 4).

### 3.3. Relations between Low Dry Matter Content and Microbiological Load of Feeds

**Figure 4:**
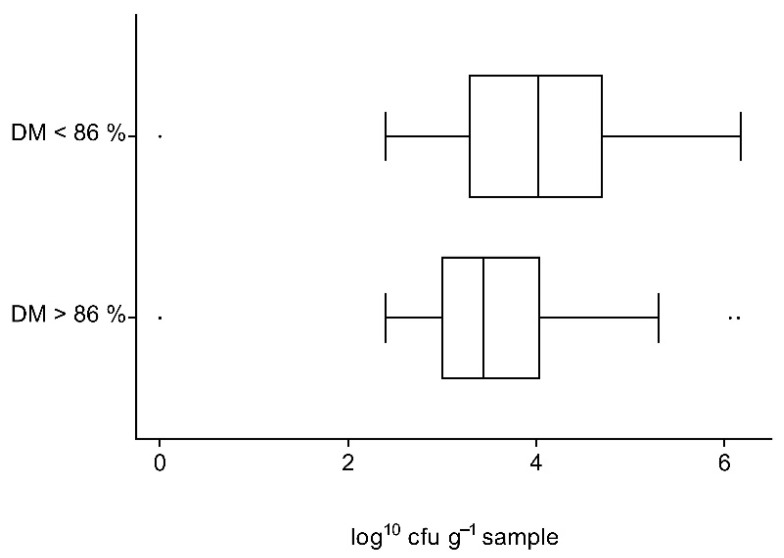
Distribution of mold counts (log^10^ cfu g^−1^ sample) in grain samples with dry matter contents of ≥86% and <86%.

The proportion of concentrates with exceeding counts of aerobic bacteria, molds or yeasts was not higher in concentrates with DM contents below 86% than in samples with ≥86% DM. Calculations were made separately for the different concentrate types. The distribution of mold counts in grains with normal or low DM contents are shown in Figure 4.

Average counts of molds in grain samples with low DM contents were significantly higher than those of grain samples with adequate values (*p* = 0.0201). Furthermore, mold count exceedances occurred significantly more frequently in samples whose DM content was low (23.1% when DM < 86% vs. 5% when DM ≥ 86%, *p* = 0.0023). No significant influence of DM content on microbiological quality could be shown for counts of yeasts or bacteria or for compound concentrates.

### 3.4. Associations of Microbiological Quality and Occurrence of Disease Symptoms Reported by Sender

Regarding the frequency of microbial counts exceeding VDLUFA orientation values [14], no significant differences could be found between samples submitted due to the occurrence of gastrointestinal disorders, elevated activity of liver enzymes or coughing and the control group (routine examinations).

Microbial deviations, regardless of the type of microorganism, occurred most frequently in samples submitted for routine examination and least frequently in samples that were submitted to clarify the occurrence of coughing (*p* = 0.3188, Table 5). Counts of aerobic bacteria were assessed as inadequate in 30.5% of samples with preliminarily reported gastrointestinal disorders (GIDs) and in 29.4% of the control group (*p* = 0.9065). Deviations regarding molds occurred in 3.8% of GIDs and 2.8% of control samples (*p* = 0.7794), whereas yeast counts exceeded VDLUFA orientation values in 10.9% of GIDs and 5.7% of control samples (*p* = 0.2986). Inadequacies in LPS contents were not significantly different in the compared groups (e.g., 37.9% of GIDs and 33.3% of control samples, *p* = 0.1955).

No significant differences were observed between samples with anamnestic reports of alteration of liver enzymes and the control group regarding counts of aerobic bacteria (21.2% and 29.4%, *p* = 0.4406), molds (7.1% and 2.8%, *p* = 0.3682) and yeasts (12.2% and 5.7%, *p* = 0.2040). Additionally, inadequate LPS contents occurred in 29.4% of samples with reported liver enzyme alteration and 33.3% of routine examination samples (*p* = 0.9047).

The frequencies of inadequate colony forming units of molds and LPS contents were not significantly different in samples submitted for clarifying the occurrence of coughing (coughing group) and samples submitted for routine examination (control group). Exceedances of VDLUFA orientation values for molds occurred in 4.2% of the coughing group and 2.8% of the control group (*p* = 0.644). LPS contents above 50 µg were determined in 30% of coughing group samples and 33.3% of control samples (*p* = 0.8929). However, we found that mold contamination differed between the preliminary reports of “coughing” and “routine examination” (*p =* 0.0313, Table 6).

Mean counts of *Aspergillus species* in the routine examination group and coughing group were compared, but the difference was not significant (*p* = 0.0812, Table 6). No differences were found in LPS levels of the concentrates depending on the preliminary report.

Microbiological quality of samples with the preliminary reports of “elevated activity of liver enzymes”, “gastrointestinal disorders (GID)” and “poor performance” did not differ significantly from samples submitted for routine examination.

## 4. Discussion

Regarding the overall microbiological quality of concentrates, deviations occurred most frequently in aerobic bacteria (26.1% of samples). Mold and yeast counts were assessed as inadequate in less than 10% of the samples (8.1% and 9.6%, respectively). When compared to forage samples, the authors found deviations regarding aerobic bacteria in 23.5%, regarding molds in 25.3% and regarding yeasts in 10.1% of forage samples. All in all, exceedance of VDLUFA orientation values occurred more frequently in forage than in concentrate samples (25.3% vs. 8.1%).

Although average microbiological counts were significantly lower in compound feeds than in grains, the frequency of germ and yeast counts exceeding orientation values of the VDLUFA [14] was higher in compound feeds compared to grains. Orientation values established by the VDLUFA represent usual contents of microorganisms in different feed types [59] and are about 50 to 100 times higher for cereals than for compound feeds (e.g., field-typical bacteria: 50 × 10^6^ cfu g^−1^ for oats vs. 0.5 × 10^6^ cfu g^−1^ for compound feed) [14]. Therefore, compound concentrates for horses have been shown to deviate from the average quality more frequently than grains for horses.

Wolf et al. [7] found that deviations in microbial counts occurred more frequently in cereals than in compound feed. In this previous study, the share of compound feed samples whose germ counts exceeded normal counts was 21%, whereas this share was 36% for cereals. Deviating mold counts occurred in 10% of compound feed and 31% of cereal samples, and deviations regarding yeasts in 34% of cereal and 9% of compound feed samples. Average microbial counts were not investigated in their study. Samples were assessed according to Meyer et al. [60], whose reference values were derived from the VDLUFA orientation values of 2002 [31]. In 2002, orientation values for mixed feeds for horses had not yet been established. For species other than horses, the threshold is higher regarding all types of microorganisms. For example, counts of spoilage molds in mixed feeds for piglets were considered normal up to 2 × 10^4^ cfu per gram sample in the year 2002 [31], whereas this threshold was set as 6 × 10³ cfu in mixed feeds for horses in the year 2017 [14]. Therefore, the assessment of microbiological deviations in compound feed was derived from different orientation values in both these aforementioned studies, which is why they can hardly be compared. The same applies when comparing our findings with those of Sliwinsky et al. [8]. They used reference values from a study by Schmidt [11], who considered germ counts above 2 × 10^6^ cfu and mold counts above 1 × 10^4^ cfu as increased and did not differentiate between types of microorganisms. Additionally, the same thresholds were used for grains and mixed feed. Sliwinsky et al. [8] found exceedances in microbial counts in cereals more frequently than in mixed feeds as well.

Microbial counts were assessed as inadequate more frequently in non-pelleted compound feeds than in pelleted feeds (*p* = 0.0055). As proposed by the VDLUFA [14], all compound feed samples, pelleted and non-pelleted, were assessed in accordance with orientation values for pelleted compound feedstuffs for horses. Since exposure to heat and pressure during the pelleting process reduces the initial load of microorganisms [17], differences in microbial load between compound feedstuffs that are not or only partly pelleted (such as muesli) are to be expected. This may also explain the fact that deviations in microbiological quality occurred more frequently in compound concentrates than in grains, since VDLUFA orientation values for pelleted feedstuffs may not be suitable for non-pelleted mixed feeds. The determination of specific reference values for compound, non-pelleted feedstuffs for horses would be desirable, since the results of this study indicate that both groups, pelleted and non-pelleted compound feeds, are different regarding microbiological quality.

Regarding the effect of oat rolling on the hygienic status, we found that average counts of yeasts and bacteria were significantly higher in rolled oats, whereas the frequency of inadequacies was not significantly higher. Mechanical damage to grains is known to impair storability [61] and be conducive to the entry and proliferation of storage molds [62]. In the studies regarding hygienic quality of equine feedstuffs mentioned above, rolled and intact oats were not investigated separately [7,8,10]. Vandenput et al. [50] compared aeroallergen contents of different feeds and bedding materials for horses and found that dust contents as well as spore contents of *Aspergillus fumigatus* were significantly higher in rolled oats than in hay of sufficient quality and considered rolled oats as a potential source of aeroallergens. Counts of *Aspergillus species* were not higher in rolled oats than in intact oats in our study (*p* = 0.5706), and neither were average mold counts (*p* = 0.3376) or LPS contents (*p* = 0.3262). In a previous study [63], it was shown that the rolling of oats increases the dust emission of the feed. In the present study, dust emissions from concentrate samples were not investigated. All in all, the hygienic quality was better in the intact than in the processed oats, but due to the results of our study no recommendation can be made regarding the feeding of horses that are sensitive to aeroallergens. For future investigations, inclusion of data on dust emissions of the different concentrates would be desirable.

The comparison of the two investigation periods showed that the hygienic quality of compound feeds was constant, whereas the deviation frequency of cereals regarding aerobic bacteria decreased and deviation frequency regarding molds increased from the first to the second period. Identical threshold values were used in both investigation periods. It has to be taken into account that cereals are mostly obtained directly from agricultural production [5], and thus, no hygienization comparable with pelletizing takes place after harvest. Due to this, cereals may be more susceptible to variations in hygienic quality. For example, changing weather conditions during the growing season can influence the extent of colonization with fungi such as *Fusarium* [64]. To the authors’ knowledge, no scientific evidence concerning the effect of weather conditions on colonization of feedstuffs with bacteria is available. Since data on date and location of growing and harvesting of the cereal samples were not available, relations between weather conditions and microbiological quality could not be investigated here.

In our study, we found that mold counts of cereal samples with DM contents below 86% were significantly higher than in samples with at least 86% DM (*p* = 0.0201). The same did not apply to compound feedstuffs. High moisture contents are known to favor microbial growth, and thus, the spoilage of feedstuffs [17]. The lack of evidence of favoring spoilage due to low DM contents in compound feed may again be attributable to the positive effects of processing on feed hygiene of compound feedstuffs. However, feedstuffs that have been processed hydrothermally can be reinfected by microorganisms under inadequate storage conditions [17]. Data on the storage conditions of the feedstuffs investigated were not available for this study.

Regarding associations between microbial contamination and reported disease symptoms, it was found that average mold counts were significantly higher in concentrate samples submitted to clarify the cause of coughing in horses than in samples submitted for routine examination (*p* = 0.0313). On the one hand, inhaling mold spores is known to induce coughing in sensitive horses [45]. On the other hand, the exceedance of mold counts occurred less frequently in concentrate samples than in forage samples of a previous study [65]. Furthermore, Vandenput et al. [50] indicated that concentrates (except for rolled oats) play a minor role as a source of aeroallergens in dust, since the emission of dust is low. Dust emission was shown to be lower in pelleted feedstuffs than in grains and lower in pelleted than in non-pelleted compound feedstuffs [63]. This suggests that the contribution of concentrates to dust contamination in horse stables may depend on the type of concentrate used. The results of the present study give further indications that concentrates may play a role in the emission of airborne allergens, since average mold counts were significantly higher in concentrates that were suspected of inducing coughing. The hygienic quality of non-pelleted compound feeds such as muesli should receive more attention, since quality differences of pelleted materials were observed in a previous study regarding dust emission [63] and in the present study regarding microbiological quality (Table 3). It has to be emphasized that, due to the retrospective character of this study, no standardized examination of clinical signs in horses could be performed. Furthermore, the examination of feedstuffs was not performed for the purpose of this study, but reports of the examination of feed hygiene were evaluated secondarily. Thus, the limitations of the present study must be taken into account. Further research would be desirable on the possible harmful effects of concentrates of minor hygienic quality on horse health.

## Figures and Tables

**Table 1:** Sensory analysis of grains and mixed feeds according to Kamphues [55].

Parameter	Acceptable	Inadequate
Texture	Dry	Clammy, wet, sticky, clumping
Odor	Product-typical	Musty, vapid, moldy, roasted, yeasty, sweet, dusty, foreign smell
Content of dirt	Unremarkable	Abrasion, dust, husks, contamination with soil/sand, admixtures (husks, feces), storage pests (insects, mites)
Color	Typical color	Grey, black-brownish, green, reddish (grains only)
Size/Form	Unremarkable	Macroscopically visible deposits, incomplete evolution of grains, swollen pellets
Integrity (grains only)	Unremarkable	Broken grains, eroded, swollen
Loupe view	Unremarkable	Deficient integrity of grains, deposits of dirt/mold, fine particles (insects, mites, visible mold growth)

**Table 2:** Orientation values for microbial counts of different concentrates in accordance with VDLUFA [14].

Type of Micro Organisms	Classification	Group No. ^1^	Exemplary Species	Orientation Value for Colony Forming Units per Gram Feedstuff		
				Oats	Barley	Mixed feed
Aerobic bacteria	Product-typical bacteria	1	*Pantoea* *agglomerans*, *Pseudomonas*, *Enterobacteriaceae*	50 × 10^6^	20 × 10^6^	0.5 × 10^6^
	Spoilage bacteria	2	*Bacillus*, *Staphylococcus*, *Micrococcus*	1 × 10^6^	1 × 10^6^	0.5 × 10^6^
		3	*Streptomyces*	0.05 × 10^6^	0.05 × 10^6^	0.01 × 10^6^
Molds	Product-typical molds	4	*Alternaria, Acremonium, Fusarium*	200 × 10^3^	40 × 10^3^	2 × 10^3^
	Spoilage molds	5	*Aspergillus* spp., *Penicillium*, *Scopulariopsis*	50 × 10^3^	30 × 10^3^	6 × 10^3^
		6	*Mucorales*	2 × 10^3^	2 × 10^3^	1 × 10^3^
Yeasts		7	All species	200 × 10^3^	100 × 10^3^	5 × 10^3^

^1^ In accordance with classification by VDLUFA [14].

**Table 3:** Proportional frequency of deviations in hygienic quality regarding dry matter content (DM) and microbiological quality (assessed using VDLUFA orientation values [14]).

Feed Type	*n*	DM (% < 86%)	Deviations Regarding Microbiological Quality (% cfu > n.c.)			
			Microbiology (Total)	Aerobic Bacteria	Molds	Yeasts
Concentrates (total)	517	18.2	28.2	26.1	8.1	9.6
Grains	273	16.9	22.6	19.3	7.2	4.5
Oats (intact)	181	13.4	19.2	16.2	6.5	6.1
Oats (rolled)	56	26.1	28.9	25.6	4.7	2.3
Other grains	36	33.3	30.8	25.0	15.4	0
Compound feed	244	19.7	38.4	38.7	11.5	17.5
Non- pelleted ^1^	151	24.3	45.1	44.6	11.8	22.6
Pelleted ^2^	93	11.8	20.5	21.1	4.8	7.7

cfu—colony forming units per gram sample; n.c.—normal counts according to VDLUFA [14]; ^1^ non-pelleted compound feed; ^2^ pelleted compound feed.

**Table 4:** Average microbial plate counts and proportional frequency of deviations in hygienic quality of cereals regarding dry matter content (DM) and microbiological quality (assessed using VDLUFA orientation values [14]) in investigation periods 1 and 2 (1993–1999 and 2010–2016).

Type of Micro Organism	*n* (Total)	Period 1 ^1^			Period 2 ²			Level of Significance (Period 1 vs. 2)	
		*n* ^1^	Log^10^ cfu (Mean)	% cfu >n.c.	*n* ²	Log^10^ cfu (Mean)	% cfu >n.c.	*p* (Mean cfu)	*p* (% cfu >n.c.)
Aerobic bacteria	197	136	6.78	23.5%	61	6.15	9.8%	0.0003	0.0243
Molds	208	141	3.20	6.4%	67	3.58	9.0%	0.0360	0.5690 ^1^
Yeasts	200	136	5.45	3.7%	64	5.11	6.3%	0.1580	0.4711 ^1^

^1^ 1993–1999; ² 2010–2016; cfu—colony forming units per gram sample; n.c.—normal counts according to VDLUFA [14].

**Table 5:** Proportional frequency of deviations in microbiological quality (assessed using VDLUFA orientation values [14]) of concentrates with different preliminary records given for submitted samples.

Preliminary Report	*n*	Proportional Frequency of Deviations (%)				
		Microbiology (Total) ^1^	Aerobic Bacteria ^1^	Molds ^1^	Yeasts ^1^	LPS ²
Routine examination	47	27.8	29.4	2.8	5.7	33.3
GIDs	165	31.3	30.5	3.8	10.9	37.9
Liver enzymes	75	21.8	21.2	7.1	12.2	29.4
Coughing	32	16.7	15.8%	4.2	0	30.0

^1^ Share of samples with microbial counts above VDLUFA orientation values [14]; ² share of samples with LPS contents of above 50 µg per gram sample [44].

**Table 6:** Quantitative determination (log^10^ cfu g^−1^ as fed) of mold counts in concentrate samples without and with occurrence of coughing after feeding (*p* = 0.0313).

Preliminary Report	*n*	Mold Counts (log^10^ cfu g^−1^)				
		Mean	s.d.	s.e.	Min	Max
Molds (total)						
Routine examination	37	2.40 ^a^	1.59	0.26	0.00	4.54
Coughing	24	3.29 ^b^	1.09	0.22	0.00	6.15
*Aspergillus* sp.						
Routine examination	37	1.12	1.59	0.26	0.00	4.30
Coughing	24	1.85	1.54	0.31	0.00	4.15

^a,b^ Different superscripts indicate significant differences (*p* < 0.05) depending on preliminary report. cfu g^−1^—colony forming units per gram sample; s.d.—standard deviation; s.e.—standard error.

## Data Availability

Data supporting the reported results can be found in the archive of examination reports of the Institute for Animal Nutrition, University of Veterinary Medicine Hannover, Foundation, Hannover, Germany.

## References

[1] Lindberg J. (2013). Feedstuffs for horses. Equine Applied and Clinical Nutrition.

[2] Meyer H., Coenen M. (2014). Pferdefütterung.

[3] Hill J. (2007). Impacts of nutritional technology on feeds offered to horses: A review of effects of processing on voluntary intake, digesta characteristics and feed utilisation. Anim. Feed. Sci. Technol..

[4] Van Saun R.J. (2006). Feed Additives, Nutritional Supplements and Nutraceuticals for Horses. Ph.D. Thesis.

[5] Douglas C.D. (2002). Quality Improvement in Oat. J. Crop Prod..

[6] Meyer H., Heckotter E., Merkt M., Bernoth E., Kienzle E., Kamphues J. (1986). Current problems in veterinary advice on feeding. 6. Adverse effects of feeds in horses. Dtsch. Tierarztl. Wochenschr..

[7] Wolf P., Kloetzer P., Paulus C., Kamphues J. (2009). A survey on the hygienic standard of feeds for horses and its implication for environmental conditions and animal health. Proceedings of the Sustainable Animal Husbandry: Prevention Is Better than Cure, Volume 2. Proceedings of the 14th International Congress of the International Society for Animal Hygiene (ISAH).

[8] Sliwinsky H., Krabisch P., Rosenberger E., Schwarz F. (2005). Hygienic quality of different forages and concentrates for horses. Pferdeheilkunde.

[9] Kamphues J. (2005). A systematic approach to evaluate the hygienic quality of feedstuffs for horses. Pferdeheilkunde.

[10] Stickdorn T., Ellis A., Kienzle E. (2012). Horse feed hygiene evaluation with microbial and sensory examination. Forages and Grazing in Horse Nutrition.

[11] Schmidt H. Mikrobiologische Richtwerte für die Futtermittelbeurteilung. Proceedings of the VII Int Kongreß für Tierhygiene.

[12] Kaya G., Sommerfeld-Stur I., Iben C. (2009). Risk factors of colic in horses in Austria. J. Anim. Physiol. Anim. Nutr..

[13] Meyer H., Kamphues J., Schneider D., Leibetseder J. (1999). Supplemente zu Vorlesungen und Übungen in der Tierernährung.

[14] VDLUFA (2017). VDLUFA Methodenbuch, Band III—Die Untersuchung von Futtermitteln.

[15] Felšöciová S., Kowalczewski P.Ł., Krajčovič T., Dráb Š., Kačániová M. (2021). Effect of Long-Term Storage on Mycobiota of Barley Grain and Malt. Plants.

[16] EU (2005). Regulation (EC) No 183/2005 OF The European Parliament and of the Council laying down requirements for feed hygiene. Off. J. Eur. Union.

[17] Kamphues J., Geor R.J., Harris P.A., Coenen M. (2013). Feed Hygiene and Related Disorders in Horses. Equine Applied and Clinical Nutrition.

[18] Boroojeni F.G., Svihus B., von Reichenbach H.G., Zentek J. (2016). The effects of hydrothermal processing on feed hygiene, nutrient availability, intestinal microbiota and morphology in poultry—A review. Anim. Feed. Sci. Technol..

[19] Magan N., Aldred D., Mylona K., Lambert R.J. (2010). Limiting mycotoxins in stored wheat. Food Addit. Contam..

[20] Neme K., Mohammed A. (2017). Mycotoxin occurrence in grains and the role of postharvest management as a mitigation strategies. A review. Food Control.

[21] Harris P.A., Ellis M., Fradinho J., Jansson J., Julliand V., Luthersson N., Santos A.S., Vervuert I. (2017). Review: Feeding conserved forage to horses: Recent advances and recommendations. Animal.

[22] Adler A. Qualität von Futterkonserven und Mikrobielle Kontamination. Proceedings of the 8th Alpine Expert Forum.

[23] Metzler A., Bauer J., Hörmansdorfer S., Pfirrmann A., Böhm R., Schneweis I., Barth D., Kamphues J., Reichmuth C. (2000). Schadorganismen und deren Stoffwechselprodukte. Potentielle Schadorganismen und Stoffe Futterm. Sowie Tier. Fäkalien: Sachstandsbericht Mitt..

[24] Mahato D.K., Devi S., Pandhi S., Sharma B., Maurya K.K., Mishra S., Dhawan K., Selvakumar R., Kamle M., Mishra A.K. (2021). Occurrence, Impact on Agriculture, Human Health, and Management Strategies of Zearalenone in Food and Feed: A Review. Toxins.

[25] Marin S., Ramos A., Cano-Sancho G., Sanchis V. (2013). Mycotoxins: Occurrence, toxicology, and exposure assessment. Food Chem. Toxicol..

[26] Benbrook C. (2005). Breaking the Mold–Impacts of Organic and Conventional Farming Systems on Mycotoxins in Food and Livestock Feed.

[27] Matthäus K., Dänicke S., Vahjen W., Simon O., Wang J., Valenta H., Meyer K., Strumpf A., Ziesenib H., Flachowsky G. (2004). Progression of mycotoxin and nutrient concentrations in wheat after inoculation with Fusarium culmorum. Arch. Anim. Nutr..

[28] Gabal M., Awad Y., Morcos M., Barakat A., Malik G. (1986). Fusariotoxicoses of farm animals and mycotoxic leucoencephalomalacia of the equine associated with the finding of trichothecenes in feedstuffs. Vet. Hum. Toxicol..

[29] Bertero A., Moretti A., Spicer L.J., Caloni F. (2018). Fusarium molds and mycotoxins: Potential species-specific effects. Toxins.

[30] Johnson P.J., Casteel S.W., Messer N.T. (1997). Effect of feeding deoxynivalenol (vomitoxin)-contaminated barley to horses. J. Vet. Diagn. Investig..

[31] VDLUFA Vorschlag: Orientierungswertschema zur Auswertung der Ergebnisse Mikrobiologischer Untersuchungen Zwecks Beurteilung von Futtermitteln Nach §7 (3) Futtermittelgesetz/Arbeitskreis Futtermittelmikrobiologie der Fachgruppe VI (Futtermittel) des Verbandes Deutscher Landwirtschaftlicher Untersuchungs- und Forschungsanstalten; 2002. https://www.lfl.bayern.de/mam/cms07/ite/dateien/31386_mikrobielle_beurteilung_von_futtermitteln_-_orientierungswertschema.pdf.

[32] Anacker G. (2006). Mikrobiologische Belastung von HauptfutterkomponentenUrsache für Gesundheitsprobleme in Milchviehherden.

[33] Kellerman T.S., Marasas W.F.O., Pienaar J., Naudé T. (1972). A Mycotoxicosis of Equidae Caused by Fusarium Moniliforme Sheldon. A Preliminary Communication. Onderstepoort J. Vet. Res..

[34] Anacker G. (2010). Mikrobiologische Futterqualität—Ursache für Gesundheitsprobleme in Milchviehherden?. Viehwirtsch. Fachtag..

[35] Leblond A., Villard I., Leblond L., Sabatier P., Sasco A. (2000). A retrospective evaluation of the causes of death of 448 insured French horses in 1995. Vet. Res. Commun..

[36] Tinker M.K., White N., Lessard P., Thatcher C., Pelzer K., Davis B., Carmel D. (1997). Prospective study of equine colic risk factors. Equine Vet. J..

[37] Voigt A., Saulez M.N., Donnellan C., Gummow B. (2009). Causes of gastrointestinal colic at an equine referral hospital in South Africa (1998–2007). J. South Afr. Vet. Assoc..

[38] Hudson J.M., Cohen N.D., Gibbs P.G., Thompson J.A. (2001). Feeding practices associated with colic in horses. J. Am. Vet. Med. Assoc..

[39] Meyer H., Ahlswede L., Pferdekamp M. (1980). Untersuchungen uber Magenentleerung und Zusammensetzung des Mageninhaltes beim Pferd. DTW Dtsch. Tierarztl. Wochenschr..

[40] Meyer H. (2001). Krampfkolik beim Pferd–Vorstellungen zu einer alimentären Genese. Pferdeheilkunde.

[41] Gieselmann A. (1994). Nutritive Anamnese bei Kolikfällen des Pferdes.

[42] Coenen M. (2013). Fütterung und Kolik. Pferdeheilkunde.

[43] Kamphues J., Böhm K. (1990). Krampfkoliken bei Pferden nach Fütterung von verdorbenem Hafer. Dtsch. Tierärztl. Wschr.

[44] Kamphues J. (1986). Lipopolysaccharide in Futtermitteln–mögliche Bedeutung, Bestimmung und Gehalte. Übers Tierernährg.

[45] Clarke A. Environmental monitoring in relation to equine respiratory disease. Current Therapy in Equine Medicine. 1992; pp. 310–315. https://www.ivis.org/library/equine-respiratory-diseases/environmental-control-of-respiratory-disease.

[46] Pirie R.S., Couëtil L.L., Robinson N.E., Lavoie J.-P. (2016). Equine asthma: An appropriate, translational and comprehendible terminology?. Equine Vet. J..

[47] Leclere M., Lavoie-Lamoureux A., Lavoie J.P. (2011). Heaves, an asthma-like disease of horses. Respirology.

[48] Bracher V., Von Fellenberg R., Winder C.N., GRUENIG G., Hermann M., Kraehenmann A. (1991). An investigation of the incidence of chronic obstructive pulmonary disease (COPD) in random populations of Swiss horses. Equine Vet. J..

[49] Pirie R.S. (2017). Severe equine asthma—an overview. Livestock.

[50] Vandenput S., Istasse L., Nicks B., Lekeux P. (1997). Airborne dust and aeroallergen concentrations in different sources of feed and bedding for horses. Vet. Q..

[51] Vandenput S., Duvivier D.H., Votion D., Art T., Lekeux P. (1998). Environmental control to maintain stabled COPD horses in clinical remission: Effects on pulmonary function. Equine Vet. J..

[52] Vesonder R., Haliburton J., Stubblefield R., Gilmore W., Peterson S. (1991). Aspergillus flavus and aflatoxins B1, B2, and M1 in corn associated with equine death. Arch. Environ. Contam. Toxicol..

[53] Casteel S.W., Rottinghaus G.E., Johnson G.C., Wicklow D.T. (1995). Liver disease in cattle induced by consumption of moldy hay. Vet. Hum. Toxicol..

[54] Anacker G. (2007). Mikrobiologische Belastung von Hauptfutterkomponenten—Ursache für Gesundheitsprobleme in Milchviehherden.

[55] Kamphues J., Wolf P., Coenen M., Eder K., Iben C., Kienzle E., Liesegang A., Männer K., Zebeli Q., Zentek J. (2014). Beurteilung von Futtermitteln. Supplemente zur Tierernährung für Studium und Praxis.

[56] Visscher C., Mischok J., Sander S., Schmicke M., Peitzmeier E.-U., von dem Busche I., Rohn K., Kamphues J. (2018). Nutrient digestibility, organ morphometry and performance in vaccinated or non-vaccinated Lawsonia intracellularis infected piglets. BMC Vet. Res..

[57] Commission, E (2009). Regulation No. 767/2009/EC of 13 July 2009 on the placing on the market and use of feed, amending European Parliament and Council Regulation (EC) No. 1831/2003 and repealing Council Directive 79/373/EEC, Commission Directive 80/511/EEC, Council Directives 82/471/EEC, 83/228/EEC, 93/74/EEC, 93/113/EC and 96/25/EC and Commission Decision 2004/217/EC. Off. J..

[58] Klötzer P. (2013). Der Mikrobiologisch-Hygienische Status Eingesandter Futtermittel für Pferde. https://elib.tiho-hannover.de/servlets/MCRFileNodeServlet/etd_derivate_00000778/kloetzerp_ss13.pdf.

[59] ADLER A. Orientierungswerte für Keimzahlen in Heu. Proceedings of the ALVA-Tagung.

[60] Meyer H. (2004). Supplemente zu Vorlesungen und Übungen in der Tierernährung. Schlütersche. https://www.amazon.de/Supplemente-zu-Vorlesungen-%C3%9Cbungen-Tierern%C3%A4hrung/dp/3794402235.

[61] Valmor Ziegler and Ricardo Tadeu Paraginski and Cristiano Dietrich, F (2021). Grain storage systems and effects of moisture, temperature and time on grain quality—A review. J. Stored Prod. Res..

[62] Magan N., Hope R., Cairns V., Aldred D. (2003). Post-harvest fungal ecology: Impact of fungal growth and mycotoxin accumulation in stored grain. Epidemiology of Mycotoxin Producing Fungi.

[63] Garlipp F., Hessel E.F., Weghe H. (2009). Airborne particle generation from horse feeds depending on type and processing. Landtechnik.

[64] Barbara Kosiak and Mona Torp and Eystein Skjerve and Birgitte, A (2004). Alternaria and Fusarium in Norwegian grains of reduced quality—A matched pair sample study. Int. J. Food Microbiol..

[65] Intemann S., Reckels B., Schubert D., Wolf P., Kamphues J., Visscher C. (2022). The Hygienic Status of Different Forage Types for Horses—A Retrospective Study on Influencing Factors and Associations with Anamnestic Reports. Vet. Sci..

